# Case Report: Circulating Anti-SARS-CoV-2 Antibodies Do Not Cross-React With Pemphigus or Pemphigoid Autoantigens

**DOI:** 10.3389/fmed.2021.807711

**Published:** 2021-12-20

**Authors:** Michael Kasperkiewicz, Marta Bednarek, Stefan Tukaj

**Affiliations:** ^1^Department of Dermatology, Keck School of Medicine, University of Southern California, Los Angeles, CA, United States; ^2^Department of Molecular Biology, Faculty of Biology, University of Gdańsk, Gdańsk, Poland

**Keywords:** SARS-CoV-2, COVID-19, autoimmune blistering diseases, ELISA, molecular mimicry

## Abstract

It is hypothesized that SARS-CoV-2 has the potential to elicit autoimmunity due to molecular mimicry between immunogenic proteins of the virus and human extracellular molecules. While *in silico* and *in vitro* evaluation of such immune cross-reactivity of human antibodies to SARS-CoV-2 proteins with several different tissue antigens has been described, there is limited information specifically pertaining to the immunological effects of COVID-19 and vaccines against SARS-CoV-2 on the development of autoimmune bullous diseases (AIBDs). Twelve seropositive post-COVID-19 individuals and 12 seropositive healthy volunteers who received two doses of the mRNA COVID-19 vaccine from Pfizer-BioNTech have been included in this case series investigation. Serum samples of these blood donors were tested for autoantibodies to the main immunobullous autoantigens, i.e., desmoglein 1, desmoglein 3, envoplakin, BP180, BP230, and type VII collagen. Our study revealed that none of the 24 anti-SARS-CoV-2 IgG-positive subjects had concomitant antibody reactivity with any of the tested autoantigens. These results argue against a relationship between SARS-CoV-2 infection/vaccines and AIBDs with respect to disease-triggering antibody cross-reactivity.

## Key Point

A link between COVID-19 or vaccines against SARS-CoV-2 and the evolution of autoimmunity has been proposed. Here, we found no evidence of an immune cross-reactivity between anti-SARS-CoV-2 protein antibodies and the major target autoantigens of pemphigus and pemphigoid.

## Introduction

A link between COVID-19 or newly developed vaccines against SARS-CoV-2 and the evolution of autoimmunity has been proposed, although the molecular mechanisms underlying these putative associations and the risk factors predicting the onset of autoimmune diseases following infection or vaccination are not well-understood (1, 2). Recently, reaction of human antibodies to SARS-CoV-2 proteins with several different tissue antigens has been described, suggesting that this molecular mimicry-based serological cross-reactivity may at least partly be responsible for the multi-organ system disorder found in some patients with COVID-19 (2).

So far, there is only limited information specifically pertaining to the effects of COVID-19 and vaccines on the development of autoimmune bullous diseases (AIBDs) (3–5). Therefore, we sought to determine, for the first time, whether immune reactivity also occurs between anti-SARS-CoV-2 protein antibodies and the main target autoantigens of pemphigus and pemphigoid.

## Case Series

Twelve post-COVID-19 individuals and 12 healthy volunteers immunized with two doses of the mRNA COVID-19 vaccine from Pfizer-BioNTech, who were all part of a previous study cohort reported by Mantej et al. (6), have been included in this investigation (Table 1). Serum samples of these blood donors were tested for autoantibodies to desmoglein 1, desmoglein 3, envoplakin, BP180, BP230, and type VII collagen. The presence of anti-SARS-CoV-2 antibodies and these pemphigus/pemphigoid antibodies was analyzed by anti-SARS-CoV-2 enzyme-linked immunosorbent assay (ELISA) (IgG) kits and a multi-variant Dermatology Profile ELISA (all from Euroimmun, Germany), respectively. Usage of human biological material was approved by the bioethics committee of the regional medical chamber in Gdańsk (Poland), and written informed consents were obtained in accordance with the Declaration of Helsinki.

**Table 1:** Characteristics of anti-SARS-CoV-2 IgG-positive subjects.

**No**.	COVID-19 vaccine^*^	**Anti-SARS-CoV-2 S1 IgG**	**Anti-SARS-CoV-2 NCP IgG**	**COVID-19 symptoms**
1	–	+	+	+
2	–	+	+	+
3	–	+	+	+
4	–	+	+	+
5	–	+	+	+
6	–	+	+	+
7	–	+	+	+
8	–	+	+	+
9	–	+	+	+
10	–	+	+	+
11	–	+	+	+
12	–	+	+	–
13	+	+	–	–
14	+	+	–	–
15	+	+	–	–
16	+	+	–	–
17	+	+	–	–
18	+	+	–	–
19	+	+	–	–
20	+	+	–	–
21	+	+	–	–
22	+	+	–	–
23	+	+	–	–
24	+	+	–	–

* *Anti-SARS-CoV-2 antibodies directed to the S1 domain of the viral spike protein and/or nucleocapsid protein (NCP) were analyzed by commercially available anti-SARS-CoV-2 ELISA kits. Eleven out of 12 non-vaccinated, seropositive post-COVID-19 individuals reported at least one of the typical COVID-19 symptoms (e.g., fever, cough, fatigue, muscle/body aches, headache, loss of taste/smell, or sore throat) that appeared in the last 12 weeks prior to blood sampling for the serological analyses. Vaccinated individuals were monitored for the presence of anti-SARS-CoV-2 IgG within 3–5 weeks of the last dose of the vaccine*.

Our examination revealed that none of the 24 anti-SARS-CoV-2 IgG-positive subjects had concomitant antibody reactivity with any of the six tested autoantigens.

These results together with a recent related report on heat shock protein autoantibodies argue against a relationship between SARS-CoV-2 infection/vaccines and AIBDs with respect to disease-triggering antibody cross-reactivity, as previously hypothesized (1, 6). Our findings also encourage COVID-19 vaccination in patients with AIBDs, as previously recommended (7). Nevertheless, it cannot be excluded that the infection or immunization may possibly induce or aggravate autoimmunity in genetically predisposed persons by alternative modalities such as non-specific bystander activation of immune cells. Further experimental approaches, including epitope mapping studies, are required to confirm our preliminary results as well as to clarify whether and how COVID-19 or respective vaccinations may potentially drive AIBDs.

## Limitations and Strengths

Our study has some limitations. For instance, long-term follow-up observations are required to prove the immunological effects of COVID-19 vaccination (both mRNA and viral vector) and infection on the development of AIBDs in a larger cohort. However, although *in silico* sequence alignment analyses and *in vitro* evaluations of cross-reactivity of anti-SARS-CoV-2 antibodies with several different tissue antigens have been previously described, we are not aware of any other study focusing on potential cross-reactivity between naturally generated SARS-CoV-2 IgG and pemphigus/pemphigoid autoantigens *in vivo*.

## Data Availability Statement

The original contributions presented in the study are included in the article/supplementary material, further inquiries can be directed to the corresponding author/s.

## Ethics Statement

The studies involving human participants were reviewed and approved by Bioethics Committee at Regional Medical Chamber in Gdańsk, Poland. The patients/participants provided their written informed consent to participate in this study.

## Author Contributions

MK and ST: study design and conceptualization, supervision, original draft preparation, and data interpretation and critical revision of the manuscript. ST and MB: analysis. All authors contributed to the article and approved the submitted version.

## Funding

This study was financed by the Polish National Science Centre (Narodowe Centrum Nauki), project numbers: 2017/25/B/NZ6/00305 and 2020/39/B/NZ6/00357 to ST.

## Conflict of Interest

The authors declare that the research was conducted in the absence of any commercial or financial relationships that could be construed as a potential conflict of interest.

## Publisher's Note

All claims expressed in this article are solely those of the authors and do not necessarily represent those of their affiliated organizations, or those of the publisher, the editors and the reviewers. Any product that may be evaluated in this article, or claim that may be made by its manufacturer, is not guaranteed or endorsed by the publisher.
